# Effect of Rickettsial Toxin VapC on Its Eukaryotic Host

**DOI:** 10.1371/journal.pone.0026528

**Published:** 2011-10-27

**Authors:** Gilles Audoly, Renaud Vincentelli, Sophie Edouard, Kalliopi Georgiades, Oleg Mediannikov, Grégory Gimenez, Cristina Socolovschi, Jean-Louis Mège, Christian Cambillau, Didier Raoult

**Affiliations:** 1 Unité des Rickettsies URMITE, UMR CNRS 6236- IRD 198, Marseille, France; 2 AFMB, CNRS UMR 6098, Marseille, France; University of Minnesota, United States of America

## Abstract

*Rickettsia* are intracellular bacteria typically associated with arthropods that can be transmitted to humans by infected vectors. *Rickettsia* spp. can cause mild to severe human disease with a possible protection effect of corticosteroids when antibiotic treatments are initiated. We identified laterally transferred toxin-antitoxin (TA) genetic elements, including *vapB/C*, in several *Rickettsia* genomes and showed that they are functional in bacteria and eukaryotic cells. We also generated a plaque assay to monitor the formation of lytic plaques over time and demonstrated that chloramphenicol accelerates host cell lysis of *vapB/C*-containing *Rickettsia*. Whole-genome expression, TUNEL and FISH assays on the infected cells following exposure to the antibiotic revealed early apoptosis of host cells, which was linked to over-transcription of bacterial *vapB/C* operons and subsequent cytoplasmic VapC toxin release. VapC that is expressed in *Escherichia coli* and *Saccharomyces cerevisiae* or microinjected into mammalian cells is toxic through RNase activity and is prevented by dexamethasone. This study provides the first biological evidence that toxin–antitoxin elements act as pathogenic factors in bacterial host cells, confirming comparative genomic evidence of their role in bacterial pathogenicity. Our results suggest that early mortality following antibiotic treatment of some bacterial infections can be prevented by administration of dexamethasone.

## Introduction

*Rickettsia* are strict intracellular bacteria that can be transmitted to humans by arthropods and can cause fatal infections [1], [2]. *Rickettsia* are classified into the 4 following groups according to their pathogenicity, some phenotypic properties and phylogeny: the typhus group (TG), the spotted fever group (SFG), *Rickettsia canadensis* (*R. canadensis*) and *Rickettsia bellii* (*R. bellii*) [2], [3]. The TG contains *R. prowazekii*, the etiologic agent of epidemic typhus, which is transmitted by the human body louse, and *R. typhi*, a flea-borne agent of endemic typhus. The SFG, which includes approximately 25 different validated species, includes *R. conorii*, which is transmitted by ticks and causes Mediterranean spotted fever in humans [4], *R. rickettsii*, which causes a severe tick-borne infection called Rocky Mountain spotted fever [5] and *R. felis*, the agent of flea-borne spotted fever in humans [6], [7]. The other two groups represent distinct lineages of rickettsiae that are currently poorly represented among characterized isolates: *R. canadensis*, transmitted by infected ticks [8] and *R. bellii*, the earliest divergent known species of *Rickettsia*. *R. bellii* are transmitted by ticks, but their potential as a pathogen remains unknown [9].

Genomic analyses of *Rickettsia* have yielded multiple toxin–antitoxin (TA) systems, including, notably, *vapB/C* (virulence-associated protein) loci [9], [10]. Currently found in the genomes of eubacteria and archaea, these gene pairs have long been regarded as an internal system, essential for maintaining plasmids and stabilizing integrons in bacterial chromosomes [11], [12]. Moreover, TA systems have been proposed to play a role in the control of protein expression, especially during starvation periods [13], [14]. The number of TA systems appears to be high in pathogenic bacteria like *Yersinia pestis* [15]. A comparative genomic study revealed that the presence of toxin-antitoxin modules is significantly associated with high pathogenicity [16]. Finally, TA systems have been found to cause rapid death in bacteria such as *S. pneumoniae* and *E. coli* on exposure to the translation-inhibiting antibiotic chloramphenicol [17], [18].

A recent study on *Pseudomonas aeruginosa* demonstrated the role of secreted TA factor in the control of surrounding bacterial populations [19]. This seminal paper exhibited that TA may be used to control the environment of pathogenic bacteria; however, experiments showed that TA failed to penetrate mammalian and yeast cells, although these factors have a direct toxic effect on their eukaryotic producer cells [20]–[22]. Thus, the TA systems found in *Rickettsia* may be toxic to their eukaryotic host via direct cytoplasmic release, following rickettsial death. This hypothesis correlates well with observations showing that chloramphenicol has a deleterious effect on cells infected with *Rickettsia rickettsii* (which possesses a TA module [5], [23], [24]) but not on cells infected with *R. prowazekii* (which lacks a TA module [25], [26]).

In this work, we demonstrated that *vapB/C* genes constitute a functional TA module in *Rickettsia*. We developed an original plaque assay to show the deleterious effect of chloramphenicol on the eukaryotic host of TA-containing *Rickettsia*. We also exhibited that VapC toxin was toxic to eukaryotes when injected into the cytoplasm as a free toxin. Finally, we demonstrated the over-transcription of the *VapC* gene in *Rickettsia* exposed to chloramphenicol and the release of toxins into the cytoplasm of infected cells.

## Results

### Detection of TA module encoding genes in *Rickettsia* spp

Our comparative genomic work on *Rickettsia* spp. revealed more TA operons than would have been expected for intracellular bacteria (Table 1, Figure S1). The genome of *R. felis* exhibits 38 toxin-antitoxin genes, 14 of which are organized into a classical TA module, and two *VapB/C*-organized modules, *vapB/C-1* and *vapB/C-2*. The 1,552,076-base-pair-long chromosome of *R. bellii* displays [9] 23 toxin-antitoxin genes, 7 of which appear in ordered TA modules and one belongs to *vapB/C* family. In the *R. conorii* genome, we found one organized *vapB/C* module. The genomes of the TG *Rickettsia*, *R. prowazekii* and *R. typhi*, contained no TA module. Moreover, other members of the *Rickettsiales* family (*Orientia* spp., *Wolbachia* spp., *Ehrlichia* spp., *Anaplasma* spp. and *Neorickettsia sennetsu*) also appeared to lack TA modules (Table 1).

**Table 1:** Number of toxin-antitoxin genes and modules of *Rickettsiales* genomes.

*Rickettsiales*	Host/vector	TA genes	Toxin	*VapC*	TA modules
*R. prowazekii*	Louse	3	3	0	0
*R. typhi*	Flea	3	3	0	0
*R. canadensis*	Tick	6	5	1	1
*R. rickettsii*	Tick	8	6	1	1
*R. conorii*	Tick	9	6	1	1
*R. massiliae*	Tick	18	9	2	5
*R. africae*	Tick	13	7	1	4
*R. slovaca*	Tick	14	8	2	2
*R. bellii*	Tick	23	13	1	7
*R. akari*	Mite	17	10	2	5
*R. peacockii*	Tick	12	6	2	2
*R. felis*	Flea	38	23	2	14
*Orientia tsutsugamushi*	Mite	2	2	0	0
*Wolbachia* from *Culex quinquefasciatus*	Mosquito	5	4	0	0
*Wolbachia* from *Drosophila melanogaster*	Fruit Fly	10	8	0	0
*Ehrlichia chaffeensis*	Tick	4	3	0	0
*Anaplasma phagocytophilum*	Tick	3	3	0	0
*Anaplasma marginale*	Tick	3	3	0	0
*Neorickettsia sennetsu*	Trematode	3	3	0	0

**Figure 1:**
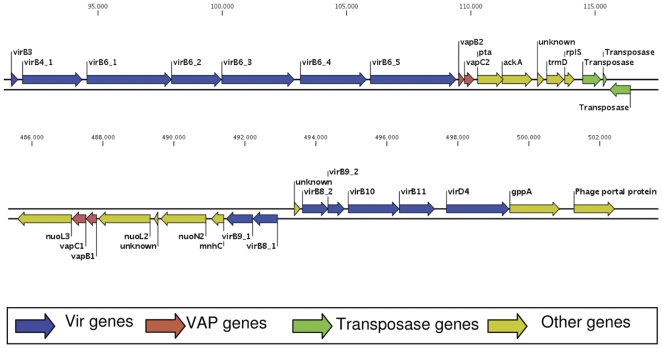
Genomic environment of the *R. felis vapBC* genes. Arrows represent the CDS strand. Red highlighting denotes the *vapBC* modules; blue denotes genes of the type IV secretion apparatus protein *VirB*; green denotes the transposase genes and yellow denotes proximal genes.

The deduced amino acid sequences of the rickettsial toxin and antitoxin revealed high homology in a BLAST query. The *R. felis* VapC-1 toxin shared 91.0% and 77.6% amino acid identity and 94% and 89% amino acid similarity with the VapC-1 of *R. conorii* and *R. bellii*, respectively, with no gap in the aligned sequences (Figure S2). Our computational approach to finding TA genes in other bacterial genomes revealed that the *vapB/C* loci that corresponds to the family of TA modules is mostly found in the genomes of cell-associated bacteria (Table 1, Figure S1). The *vapB/C* genes display characteristics of DNA segments acquired through lateral gene transfer (LGT). We found an unexpected phylogenetic tree of *vapB/C* sequences related to the core gene of *Rickettsia*, while the *R. felis vapB/C-2* module is clustered phylogenetically with the spirochetes (Figure S3, Data S1). The irregular distribution of *vapC-1* and *vapC-2* is found in a variety of *Rickettsia* species (Table 1). The local environment of the *vapB/C* modules is different across bacterial genomes. Only the two different *R. felis vapB/C* modules were found adjacent to the virB operons and close to two transposases, and in *R. akari* and *R. felis*, *vapB/C* modules were found near type IV secretion systems (T4SS). In the other *Rickettsia* spp., T4SS were found in distant regions of the chromosome *(Figure 1). Among the species that are associated with the same host arthropod, similar vapB/C modules are shared independent of the phylogenetic relationship, as is the case for R. bellii and R. conorii in tick vectors (Table 1). When combined, these results are consistent with acquisitions of vapB/C modules through LGT.*

### *Rickettsia* VapB/C controls cell growth of *E. coli* and *S. cerevisiae* by RNase activity

**Figure 2:**
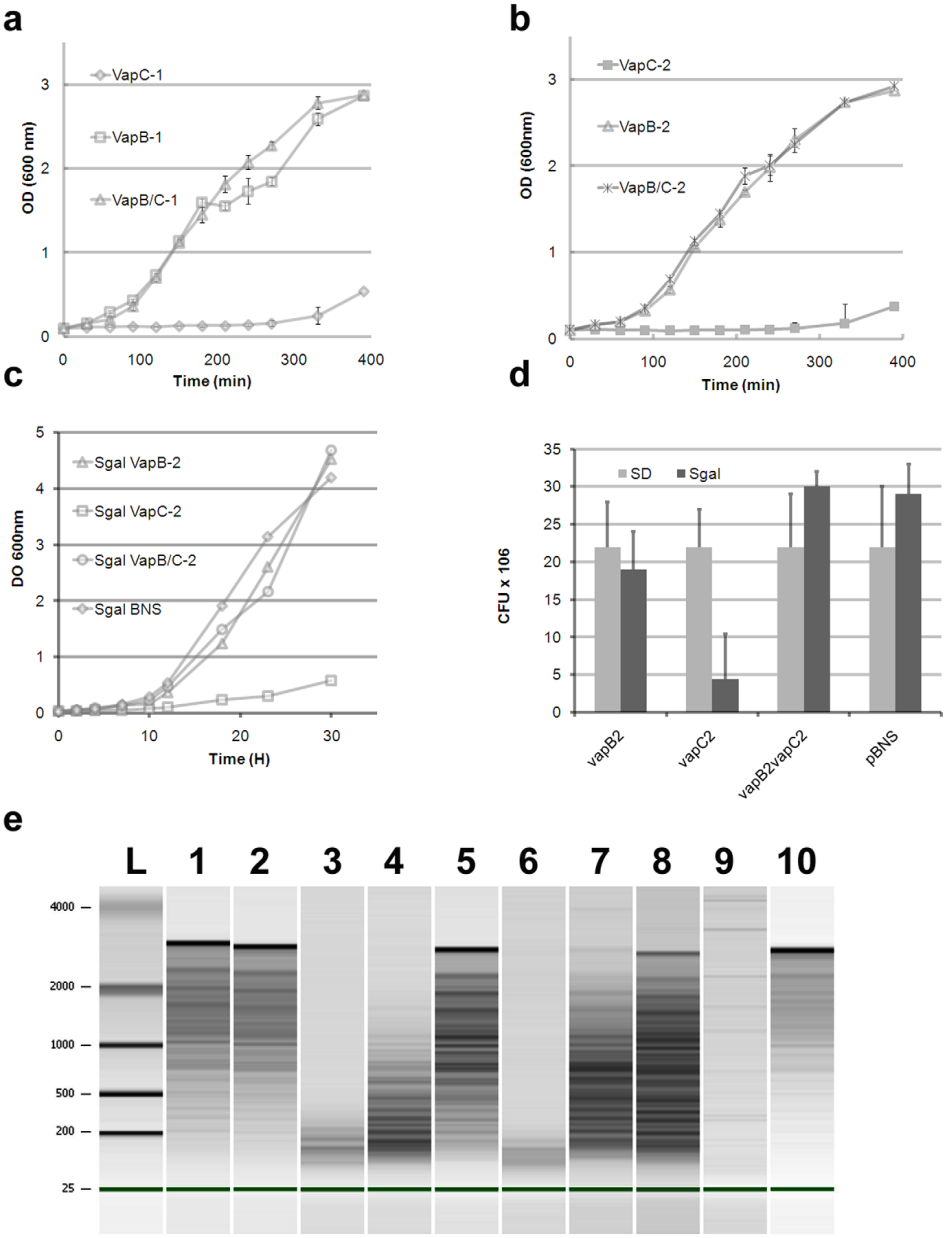
Heterologous host growth inhibition by *R. felis* VapC toxin is controlled by antitoxin VapB. **a.** Growth curves of *E. coli* cultures expressing the *vapB-1*, *vapC-1* or *vapBC-1* operon. **b.** Growth curves of *E. coli* cultures expressing the *vapB-2*, *vapC-2* or *vapBC-2* operon. The error bars represent the mean of triplicates ± standard deviation. **c.** Growth curves of yeast cells in Sgal induction media expressing *R. felis vapB-2*, *vapC-2*, VapB2-VapC2 and/or control pBNS. **d.** Yeast toxicity assays of *S. cerevisiae* harboring plasmids with indicated genes or empty vector, under inducing (Sgal) or noninducing conditions (SD). **e.** *In vitro* RNase activity of free *R. felis* and R. bellii VapC-1 proteins: The MS2 phage RNA profiles after incubation reactions with irrelevant protein (lane 1 and 2), *R. felis* VapC-1 (lane 3), *R. felis* VapB/C-1 complex (lane 4), *R.felis* antitoxin VapB-1 (lane 5), *R. bellii* VapC-1 (lane 6), *R. bellii* VapB/C-1 complex (lane 7), *R. bellii* antitoxin VapB-1 (lane 8), RNase A as positive control (lane 9), without protein (lane 10).

As is usual for fastidious and intracellular bacteria, for which transformation assays are not usually suitable, an experimental host was used to demonstrate that the rickettsial TA modules were functional. We selected the *vapB/C-1* and *vapB/C-2* genes from *R. felis* and the *vapB/C-1* gene from *R. bellii* to transform *E. coli*. We demonstrated that *vapC* gene expression in transformed bacteria induced a clear growth-inhibitory phenotype, whereas *vapB* expression did not. Moreover, the VapB antitoxin controlled the action of the VapC toxin in bacteria expressing the *vapB/C* operon (Figure 2a and 2b). Furthermore, in the yeast cells used as a second heterologous host, we found a decrease in the growth of cells expressing *R. felis vapC-2* compared with those expressing *R. felis vapB-2* or double-transformed *vapC-2/vapB-2* cells (Figure 2c, 2d). Microscopic observations of the *vapC-2* yeast morphology confirmed that yeast cells were highly susceptible to the toxin's effect. Ultrastructural abnormalities, including exaggerated vacuolation and nuclear alterations, suggested apoptotic action by the toxin (Figure S4).

The *R. felis* VapC-1 and VapC-2 toxins and the *R. bellii* VapC-1 toxin were produced in large quantities when the genes were cloned in tandem with their respective *vapB* genes in *E. coli*. We modified the 5′ end of the antitoxin gene with a sequence encoding a histidine tag to purify a native VapB/C complex. The complex and dissociated proteins were then separated by gel-filtration chromatography and used in a series of functional assays. Next, we established that the fractions corresponding to toxins and antitoxins were enzymatically active in a *in vitro* RNase assay. The free *R. felis* VapC-1 and free *R.bellii* VapC-1 recombinant proteins were able to hydrolyze the bacteriophage MS2 genomic RNA; the respective VapB-1 antitoxins had no detectable effect on RNA but counteracted the toxins RNase activity when forming a complex (Figure 2e). The free *R. felis* VapC-2 was able to hydrolyze RNA; the VapB-2 alone or in complex with VapC-2 was not (Figure S4). Thus, we showed that rickettsial VapC have RNase activity, which are controlled by VapB in the TA complex.

### Death of *Rickettsia*-hosting cells upon exposure to chloramphenicol

**Figure 3:**
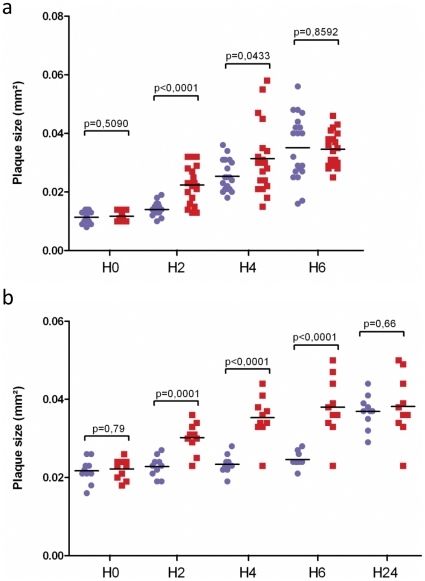
Transient increased size of PFU after chloramphenicol addition in *R. conorii* infected cultures. **a.** The mean sizes ± standard deviation (sd) of the lytic plaques in infected cells treated with chloramphenicol (red square) or mock-treated with PBS (blue circle) were monitored at 0, 2, 4 and 6 hours after treatment. **b.** The mean ± sd size of 10 lytic plaques for infected cells treated with chloramphenicol, (red square) and infected cell mock-treated with cell culture medium (blue circle). The mean ± sd areas of PFUs were processed with Student's *t*-test, and the p values are noted on each graph.

We designed a new assay to evaluate chloramphenicol action in *Rickettsia*-infected cells. We found that treatment resulted in the early death of host cells infected with *R. conorii*, *R. felis*, and *R. bellii*, but not *R. prowazekii*; this effect correlates with the presence of TA modules in the bacterial genome. The early death effect of chloramphenicol in *R. conorii*-infected cells was monitored with a plaque-forming unit (PFU) assay. Lytic plaques were formed more rapidly in infected cells treated with chloramphenicol than in the untreated cultures. The lytic plaques of infected cells treated with chloramphenicol were also significantly larger than those in untreated cells after 2 and 4 hours of incubation (p<0.0001 and p = 0.0433, respectively, Figure 3a). At 6 hours, the plaques in untreated culture reached the size of the plaques in chloramphenicol-treated culture. In a second assay, we improved the sensitivity of the test by using square Petri dishes to monitor, microscopically and individually, the size of each plaque 2, 4 and 6 hours following antibiotic testing. The chloramphenicol-treated cells exhibited early mortality marked by a significant increase in the size of plaques at each tested time, including at 6 hours (Figure 3b). The chloramphenicol blocked bacterial protein synthesis with a complete bacteriostatic effect nearly 6 hours post-treatment. The bacteria were thus prevented from spreading to uninfected neighboring cells. In the chloramphenicol-treated culture, the death of the host cell is probably independent of the infection rate and instead is triggered directly by the bacteria.

**Figure 4:**
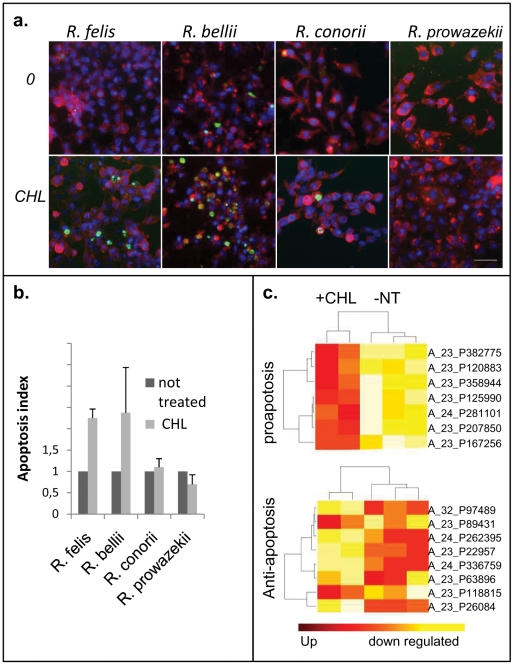
Increased apoptotic response after chloramphenicol treatment of TA–containing, *Rickettsia*-infected cells. **a.** TUNEL and indirect immunofluorescence of *Rickettsia*-infected cells to visualize the apoptotic cells (green) and bacteria (red) associated with nuclear DAPI staining (blue) in both untreated (0) and chloramphenicol-treated (CHL) cells. Scale bar represents 40 µm. Upper and lower panels of each infection were monitored with equal exposure time to compare the signal intensities. **b.** The graph represents the apoptosis ratio in each infected culture with or without chloramphenicol exposure. **c.** Clustering of differentially expressed genes of *R. felis*-infected HMEC cells in the presence of chloramphenicol (+CHL) or *R. felis*-infected HMEC cells that were not treated (NT). Differentially expressed genes associated with apoptosis of *R. felis*-infected HMEC cells (GO: 0008629) are depicted in the upper panel; those associated with anti-apoptosis processes (GO: 0006916) are in the lower panel. Red plots represent genes that are up regulated, and yellow plots represent genes that are down regulated. On the right, references regarding the differentially expressed genes related to apoptotic and anti-apoptotic pathways, as well as the significantly over-represented GO biological processes of up- and down-regulated genes, are provided (Table S1).

We confirmed early mortality in the infected cultures treated with antibiotics with a TUNEL assay of *R. conorii*-infected cultures. This assay, which indicates the presence of degraded genomic DNA, is an apoptotic end-marker. A slight but significant increase in TUNEL-positive cells was detected at 6 hours (p = 0.0005). This effect was even more obvious in the cells infected with *R. bellii* or *R. felis*, both of which have several TA systems in their genomes (see below). In these tests, the ratio of apoptotic cells to living cells was twice that of the controls without antibiotics. In contrast, cells infected with *R. prowazekii* (whose genome does not contain TA cassettes) did not undergo apoptosis following chloramphenicol treatment, despite having a high density of bacteria per cell (Figure 4a and 4b).

To identify the expressed genes that may contribute to apoptosis, we used a whole-genome approach to study *R. felis*-infected human endothelial HMEC-1 cells, which were incubated in the presence or absence of chloramphenicol. Gene-expression analysis revealed the up-regulation of 270 probes and the down-regulation of 330 others. Four gene ontology (GO) terms related to apoptosis were over-expressed (p<0.01), and 13 GO terms related to anti-apoptosis, cell division, the cell cycle and mitosis were down-regulated in chloramphenicol-treated infected cells (p<0.05, Figure 4c, 4d and Table S1). The major anti-apoptotic gene, *bcl2*, was markedly down-regulated in infected cells that were treated with antibiotics. Although *R. felis* induced a specific program in HMEC-1 that supported cell survival, the presence of chloramphenicol was sufficient to induce apoptosis in infected cells. In conclusion, chloramphenicol causes early death through apoptosis of cells infected by *Rickettsia* encoding TA cassettes.

### Dexamethasone prevents VapC-induced toxic effects of chloramphenicol

**Figure 5:**
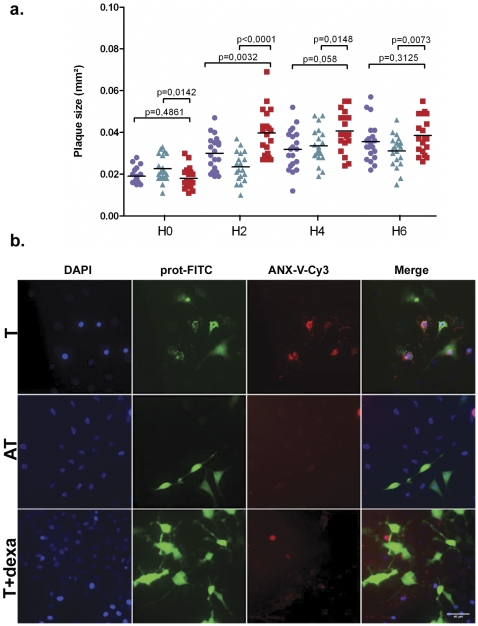
Dexamethasone treatment inhibited apoptosis induction in *R. conorii*-infected cells or in a model of cells microinjected with *R. felis VapC*-2 protein. **a.** The mean ± standard deviation (sd) of the lytic plaque sizes from infected cells that were treated with chloramphenicol (red square), pre-incubated with dexamethasone and treated with chloramphenicol (green arrowhead) or mock treated with PBS (blue circle) at 0, 2, 4 and 6 hours after treatment. **b.** Microinjected L929 cells with dextran-FITC associated with VapC-2 protein (T) or VapB-2 (AT). From left to right, all panels show fluorescence microscopy images using DAPI, dextran-FITC and Anx-V -Cy3 staining and the overlay channel. In the lower panels (T+dexa), the L929 cells were pre-incubated for 24 hours in MEM supplemented with 1 µM dexamethasone before the toxin microinjection. Scale bars represent 40 µm (see the experimental procedures section).

The protective effect of dexamethasone against toxicity in chloramphenicol-treated *R. conorii-*infected cells was monitored with a plaque-forming unit (PFU) assay. We microscopically monitored the size variations of the lytic plaques, which formed following antibiotic treatment, using L929 recipient cells [27]. The recipient cells were pre-incubated for 24 hours with or without dexamethasone. The plaques were significantly larger in the chloramphenicol-treated infected cells that were not pre-incubated with dexamethasone when compared to those that were pre-incubated with dexamethasone (2 hours, p<0.0142; 4 hours, p<0.0001, Figure 5a).

Recombinant VapC proteins are harmless in contact with the outside of eukaryotic cells, and we speculated that these molecules are not internalized. We accordingly designed a device to inject *R. felis* VapC-2 and *R. bellii* VapC-1 into eukaryotic cells. Microinjection was followed by Annexin-V staining to label the apoptotic cells and EthD-1 to measure cell injury. Microinjection of the toxin caused rapid apoptotic cell death within 2 hours, whereas most of the cells injected with the antitoxin or control remained viable at 20 hours (Figure 5b). Time-lapse video confirmed these results for *R. felis* VapC-2 and *R. bellii* VapC-1 (Video S1). Pre-incubation of cells with dexamethasone for 24 hours protected microinjected cells from toxin-induced cell death (Figure 5b). The results of cell viability and Annexin-V labeling analysis showed that pre-treatment with dexamethasone increased cell viability significantly (p<0.01) compared to cells that were not pre-treated. In conclusion, dexamethasone protected both cells infected with *Rickettsia* and treated with chloramphenicol and those subjected to cytoplasmic injection of free VapC. We suggest that dexamethasone acts to block the apoptosis program.

### Expression and release of VapC in host cell cytoplasm

**Figure 6:**
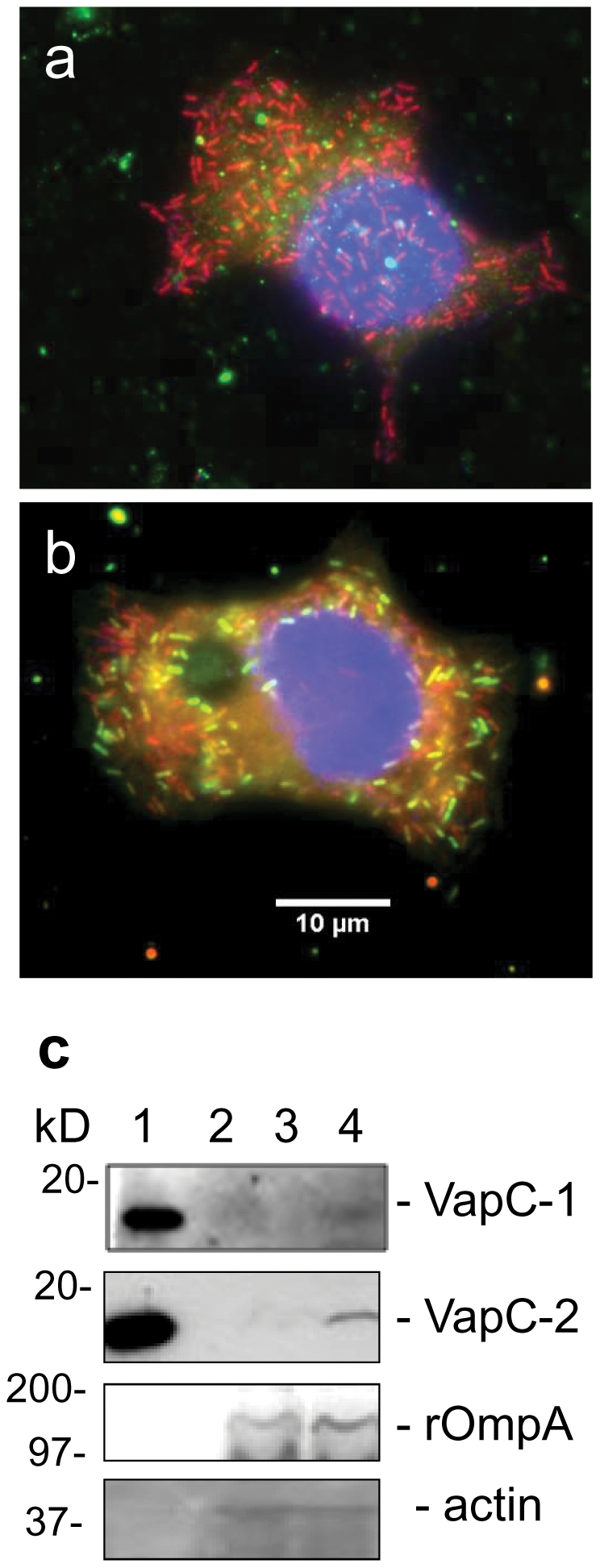
Activation of vapC modules upon chloramphenicol treatment and toxin release into the host cell cytoplasm. FISH assays of **a.** untreated or **b.** chloramphenicol-treated *R. felis*-infected cells using a universal 16S rRNA-targeting oligonucleotide probe (red), antisense *vapB/C* probes (green) and DAPI staining (blue) to visualize the nucleus. Scale bars represent 10 µm. **c.** Western blot analyses using a 15% SDS-polyacrylamide gel loaded with purified recombinant VapC1 and VapC2 toxins (lane 1) and cytoplasmic fractions of *R. felis*-infected cells without (lane 3) or with (lane 4) chloramphenicol treatment. Lane 2 is not loaded. The proteins were probed with the antisera, as indicated on the right.

To confirm the role of VapC toxins in the death of infected host cells exposed to chloramphenicol, we identified over-transcription of *vapC* modules in treated *Rickettsia* cultures by FISH analysis (Figure 6a). This effect was confirmed by RT-PCR, verifying the existence of a toxic response to antibiotic treatment. We also used a western blot analyses with polyclonal antibodies directed against VapC1 and VapC2 to show that more toxin was detected in the cytoplasmic fractions of treated infected cells than in untreated infected cell fractions (Figure 6b). Both assays confirmed that chloramphenicol causes the release of a fatal toxin in the cytoplasm of *Rickettsia*-infected cells.

## Discussion

Our report constitutes the first description of functional *Rickettsia* TA modules acquired through lateral gene transfer. Our hypothesis was that the toxic effect of *vapB/C*-containing *Rickettsia* on its host could be related to the release of toxin in host cell cytoplasm from a TA complex that is secreted or released following rickettsial death. To demonstrate this phenomenon, we established an original method for evaluating the formation of lytic plaques *in vivo* and monitoring the early changes in their size. After exposure to chloramphenicol, the *Rickettsia* with TA modules in their genomes caused the premature death of their host cells, while *Rickettsia prowazekii* (whose genome is devoid of such modules) did not. We found that VapC toxin was expressed and released in the cytoplasm following chloramphenicol exposure but was not released in untreated infected cells. When combined, these results show that *vapC* is over-transcribed and over-expressed and that VapC is released in the host cytoplasm when *Rickettsia* are exposed to chloramphenicol. To demonstrate that this toxic effect was related to VapC, we expressed the gene in *E. coli* and *S. cerevisiae* and demonstrated that the protein was toxic in both of these receptor cells and that the toxicity was related to its RNase activity. By using microinjection to introduce free toxin into the cytoplasm of L929 cells, we demonstrated that this toxin causes cell death.

After chloramphenicol exposure of infected cells or after VapC injection, the cells initiate an apoptotic program that can be prevented by dexamethasone treatment. Our data suggest that chloramphenicol is harmful specifically to cells infected with *VapB/C*-containing *Rickettsia* and that the release of this toxin in the cytoplasm upon exposure to the antibiotic causes early cell death.

The presence of TA with toxic activity in *Rickettsia* is fuel for the debate on the possible neglected role of TA in intracellular bacterial pathogenicity [16]. Indeed, TA cassettes have thus far been considered mainly in terms of the conservation of plasmids and integrons or control of bacterial growth during stressful periods [11], [12]. The recent discovery that toxin secreted by a T6SS has a role in monitoring the environment [19] suggests a role for TA in environmental regulation. It is likely that such effects will be demonstrated in extracellular bacteria; however, this hypothesis should be extended to intracellular bacteria as well. In a recent study on *Bartonella rattaustraliani*, the transfer of the plasmid that encodes both T4SS and the TA cassette was found to allow intracellular growth of *B. henselae* in amoebae. This result demonstrated a critical role for those genes in host-parasite interactions [28] and confirmed the *in silico* data on specialized obligate intracellular bacteria [29].

Finally, our data supports the hypothesis that some of the negative effects of the antibiotic treatment of rickettsioses or other intracellular bacterial infections may be caused by bacterial TA, which is released in the cytoplasm of infected cells. Such effects could be prevented by dexamethasone treatment, as proposed earlier [24]. We suggest that this phenomenon might explain why dexamethasone is protective during antibiotic treatment for some bacterial infections [30].

## Materials and Methods

### Ethics statement

No ethics statement is required for this work.

### Bacterial strains

*R. felis* California 2 strain (NC_007109), *R. conorii* Malish strain (ATCC VR-613), *R. prowazekii* Breinl strain (ATCC VR-142) and *R. bellii* RML 369-C strain (NC_007940.1; Rocky Mountain Laboratory Collection, Hamilton, Montana, U.S.A.) were cultured and used as previously described [31]. The *R. felis* genes *vapB-1* (RF_0457), *vapC-1* (RF_0456), *vapB-2* (RF_ORF0094) and *vapC-2* (RF_ORF0095) and the *R. bellii* genes *vapC-1* (RBE_1021) and *vapB-1* (RBE 1020) were amplified using genomic DNA as the template and cloned using a Gateway system (Invitrogen Ltd., Paisley, U.K.).

### Toxin-antitoxin databases and identification

Text mining searches were conducted in the GenBank protein database (Release 168) using both acronyms and the full names of the most-studied TA families: *VapB/C*, *RelE/B*, *ParE/D*, *MazE/F*, *phd/doc*, *ccdA/B* and *higA/B*. After manual curation, a BLAST database was built for the TA family. For TA identification, the protein set from a whole genome was first arranged by genome coordinates. Each protein was used in a BLASTP query of our TA databases. A hit was defined as a match to a query protein with an E-value threshold of 10^−5^, which should cover at least 70% of the TA with more than 30% identity. Lastly, the TA module was recognized if and only if two contiguous proteins in the genome were annotated as toxin and antitoxin or vice versa. A BLAST search was also used to identify T4SS in the studied species. A hit was defined as having an E-value threshold of 10^−5^, coverage of at least 70% and an identity percentage higher than 30%. The position of the T4SS on the chromosome was then determined.

### Plaque-forming unit assay

Confluent L929 cells, which were grown in MEM (Invitrogen, Paisley, U.K.) and supplemented with 4% fetal calf serum (FCS) and 2-mM L-glutamine in 60-mm Petri dishes (Falcon, Becton Dickinson, Cowley, U.K.), were infected with a 1-ml solution containing 10^2^ PFU/ml of *R. conorii*. After 1 hour of incubation at room temperature, the inoculum was removed, and the infected cells were overlaid with 5 ml of MEM that was supplemented with 4% FCS, 1% L-glutamine and 0.5% agar. The Petri dishes were incubated for 5 days at 32°C in a 5% CO_2_ incubator. After 5 days, chloramphenicol was added to a final concentration of 50 µg/ml (T0) and volume of 200 µl, and 200 µl of PBS was added as negative controls. Our study was based on the lytic area size variations. The plaques were observed with an inverted microscope (Olympus CKX41, objective ×40). We monitored the lytic plaque sizes on Petri dishes every 2 hours from 0 to 6 hours post-antibiotic exposure. We obtained images of 20 lytic plaques at different times after the addition of antibiotic or cell culture medium to Petri dishes using a camera (Nikon 995, 5 million pixels). Each image was analyzed with ImageJ software (U.S. National institutes of Health, Bethesda, MD; http://rsb.info.nih.gov/ij/) to quantify the total area of each lytic plaque. An application was used to trace the outline of each lytic plaque and calculate the area (mm^2^) using the pixels/mm^2^ ratio, as measured by a micrometer (scale bar: 0.2850 pixels/mm).

To study the protective effect of dexamethasone, the cells were pre-incubated with 1 µM dexamethasone for 24 hours before the addition of chloramphenicol (50 µg/ml). We obtained images of 20 lytic plaques at 0, 2, 4 and 6 hours for the cells pre-incubated with dexamethasone that were treated with chloramphenicol or PBS and for the cells that were treated with chloramphenicol without pre-incubation with dexamethasone. Each image was analyzed with ImageJ software.

In a second assay, we improved the sensitivity of the method using square Petri dishes to monitor, microscopically and individually, the growth of the same lytic plaques over time. The plaque assay was performed as previously described. After a 5-day incubation, chloramphenicol (50 µg/ml) and cell culture medium were added to a Petri dish, serving as a negative control. We obtained images for 10 lytic plaques at 0, 2, 4, 6 and 24 hours per square Petri dish with chloramphenicol and with cell culture medium. Each image was analyzed with ImageJ software, as described above.

The mean area and standard deviation were calculated for each assay. Each experiment was performed in triplicate. The data were analyzed using Student's *t*-test. Statistical analyses was performed using Graphpad Prism 5. Differences were considered statistically significant at p<0.05.

### TUNEL assays

Experiments were performed according to the manufacturer's instructions (TUNEL kit; Roche, Darmstadt, Germany) on 70% sub-confluent XTC2 cells infected with *R. felis* or L929 cultures infected with *R. conorii*, *R. bellii* and *R. prowazekii* at 5 bacteria/cell. All infected cultures were then incubated for 3 to 5 days in a 5% CO_2_ incubator until approximately 50% of the cells were productively infected, as visualized by May-Grünwald-Giemsa staining. TUNEL was coupled to indirect immunofluorescence using a rabbit anti-*Rickettsia* antibody (1∶1,000). After being washed with PBS, the samples were incubated with an anti-rabbit Alexa 546 (1∶300; Molecular probes, Invitrogen, Cergy-Pontoise, France). After 3 further washings, the coverslips were air-dried and mounted with 4′,6-diamidino-2-phenylindole (DAPI ) from a ready-to-use solution (ProLong Gold Antifade Reagent; Molecular Probes). The apoptotic cells were counted using ImageJ software (http://rsb.info.nih.gov/ij/ US National Institutes of Health, Bethesda, MD). The apoptosis index of the infected cells was calculated as the percentage of apoptotic infected cells over total infected cells.

### Gene expression of *R. felis*-infected human cells

Twenty-four hours after trypsinization and passaging at 70% confluence, the human microvascular endothelial cell line (HMEC-1, [36]) was infected with *R. felis* California 2 strain at a rate of 5 bacteria/cell until at least 50% were productively infected. *R. felis*-infected cultures were then treated with chloramphenicol at 50 µg/ml for 6 hours; a control group was given a mock treatment. The cells were harvested and RNA was extracted using the RNeasy Mini Kit (Qiagen, Hilden, Germany). DNA contamination was removed using a Turbo DNA-free Kit (Ambion). RNA was labeled using a Quick Amp Labeling Kit One-color (Agilent) and hybridized onto a Whole Human Genome Microarray (4×44 K; Agilent), as recommended by the manufacturer. The arrays were scanned with a DNA Microarray Scanner (Agilent), and the data were extracted using Feature Extractor (Agilent). The microarray data discussed in this publication were deposited into the NCBI's Gene Expression Omnibus [32] and are accessible through GEO (http://www.ncbi.nlm.nih.gov/geo/query/acc.cgi?acc=GSE22072), series accession number GSE22072; all data are MIAME-compliant.

### Molecular cloning and expression of *vapB* and *vapC* genes in *E. coli* and *S. cerevisiae*

The cloning of *R. felis vapB-1*, *vapC-1*, *vapB-2* and *vapC-2* genes and *R. bellii vapB-1* and *vapC-1* for expression in *E. coli* was performed after performing PCR using a Gateway system (Invitrogen, Ltd., Paisley, U.K.). The cloning procedures were performed in yeast cells by homologous recombination with flanking sequences of the plasmid vector pYes2 and pYes3 (Invitrogen, Ltd., Paisley, U.K.). The primers used in this study to amplify TA genes are listed in Table S2. The expression and protein purification procedures in *E. coli* have been previously described [33]. For the *vapBC* constructs, gel filtration was used to separate the complexes from the excess VapB and VapC; detailed procedures are described in Methods S1 [34]. The identity and integrity of all proteins was confirmed by MALDI-TOF-Mass Spect (MS) and protein identification (Data S2 and Data S3) [35].

### Microinjection of L929 cells

Solutions for microinjection contained 20 mg/ml FITC-dextran as fluorescent tracers of purified *R. felis* VapC-2, *R. bellii* VapC-1 toxins, *R. felis* VapB-2 antitoxin and the *R. bellii* VapBC-1 complex. Control injections were conducted using commercial BSA (Sigma Aldrich, L'Isle d'Abeau Chesnes, France) and fluorescent tracers in buffer; these injections served as a non-specific protein control for the calibration of microinjection experiments. The solutions were centrifuged before use (10,000 *g* for 10 min) to remove particulate matter. Microinjections were performed under a phase contrast microscope using an Eppendorf Transjector 5246 that was controlled by an Eppendorf 5170 Micromanipulator and coupled to Femtotips II needles (Eppendorf AG, Hamburg, Germany). On average, approximately 100 cells were injected at a pressure of 100–200 hPa for 0.1 s. After microinjection, the cells were returned to the incubator for an additional 2 to 24 h at 37°C or directly processed for time-lapse video analyses. The cytotoxicity of the microinjection process was measured by ethidium homodimer-1 (EthD-1; Molecular Probes, Eugene, Oregon, USA) and Annexin-V (Anx-V; AbCys SA, Paris, France) staining of labeled apoptotic cells.

### Time-lapse video on microinjected cells

The microinjection process and its resulting effects on the cells were visualized using time-lapse imaging with a microscope. The dynamics of the cell periphery, membrane blebbings and nuclear retractions that corresponded to known apoptosis markers were examined using a Nikon TE2000S microscope system equipped with an LWD 40×/0.56 PH1/APL objective for phase contrast and a super-high-pressure mercury lamp for fluorescence microscopy. The system was connected to a Sight DS-U1 digital camera and Lucia G workstation software. To record the effect of the microinjections on cell viability, images were collected at 8 images/s for 1 minute and then at 5- or 10-s intervals until the cellular processes were fully observed. In between the sequences, fluorescent images were captured to visualize the positive FITC-dextran microinjected cells. The animated image sequences were recorded and time-stamped to match the time-lapse phases (http://ifr48.timone.univ-mrs.fr/article/toxine-antitoxine/video.zip).

### Determination of VapC-2 ribonuclease activity

One microgram of total *E. coli* RNA or one µg of genomic RNA from bacteriophage MS2 (Roche, Mannheim Germany) was added to a reaction containing 50 mM Tris pH 8.0, 10 mM MgCl_2_ and 1 µg of purified protein (total reaction volume of 20 µl). RNase activity tests were conducted with VapC toxin, the VapBC complex, VapB antitoxin, 1 µg of a commercially available RNase A (10 u/µl) as a positive control, 1 µg of BSA (bovine serum albumin) as a negative control and a mock treatment with DEPC H_2_O. The reaction was allowed to proceed for 30 min at 25°C, and the samples were then purified with an RNeasy cleanup kit (Qiagen) before being subject to capillary electrophoresis using an Agilent Bioanalyzer (RNA Pico Chip).

### Detection of VapC toxins in infected cells

Western blot analyses were performed on the cytoplasmic fractions from the *R. felis*-infected XTC2 cell line [36]. The infected cells were resuspended in TE before vortexing with glass beads and then clarified by centrifugation at 1500× g for 5 min to pellet out the cellular debris and nuclei. The supernatants were centrifuged again at 15000× g for 10 min to separate the bacteria from the cytoplasmic fraction. The concentration of protein in the supernatants was determined using the Bradford protein assay (Bio Rad). Aliquots of 25 µg were diluted 1∶1 with 2× Laemmli sample buffer, heated to 95°C for 3 min, loaded onto a 15% SDS polyacrylamide gel, electro-transferred to nitrocellulose sheets and blocked with 5% non-fat dry milk (Difco) in PBS. Mouse anti-VapC-1 antibody and mouse anti-VapC-2 antibody were used at dilutions of 1∶100; mouse polyclonal antibodies for total *R. felis* or *R. conorii* and mouse monoclonal antibody to actin and secondary antibody alone were used as controls. Bound antibodies were detected using a horseradish peroxidase-conjugated goat anti-mouse antibody (1∶1000) in an enhanced chemoluminescence system with Western blot detection reagents (GE Healthcare). Quantification of the relative abundances of toxins was performed using Western blot analyses with a fluorescence-labeled secondary antibody (rhodamine-conjugated anti-mouse) and an FITC-conjugated anti-actin antibody). The Western blot image was scanned using *Typhoon* 9000 at 50-µm resolution and analyzed using the ImageQuant TL version 5.0 software (GE Healthcare).

The polyclonal antibodies against the toxins were generated by immunizing mice with a keyhole limpet hemocyanin-conjugated peptide, STKQWSHYGQTYIS or FKRIPNLILENWDK, corresponding to the N-terminus of VapC-1 or C-terminus of VapC-2, which are 100% identical in this region of each molecule.

### Fluorescence *in situ* hybridization of *vapBC* modules

The cells were fixed in 4% formaldehyde in PBS buffer (pH 7.4; 0.14 M NaCl, 2.7 mM KCl, 10.1 mM Na_2_HPO_4_ and 1.8 mM KH_2_PO_4_) at room temperature (RT) for 1 hour. The cells were washed twice in PBS and permeabilized by treatment with PBS (0.1%) triton X-100 (0.1%), tween 20 (30 min at RT), lysozyme (Sigma-Aldrich, Deisenhofen, Germany) and 5 mg/ml in TE (1 h at 37°C). After post-fixation in 4% PFA in PBS, the reactions and fixation steps were quenched with PBS-glycine (2 mg/L) incubation. Both prehybridization and hybridization were performed in a Hybridizer instrument, following the manufacturer's recommendations (Dako, Trappes France). Slides were prehybridized at 60°C for 30 min. The prehybridization solution consisted of 4× SSC (1 SSC: 15 mM sodium citrate and 0.15 M sodium chloride), 10% dextran sulfate, 1× Denhardt's solution, 0.5 mg/ml *E. coli* tRNA, 20 mM ribovanadyl complexes, 1 mM EDTA and 25% formamide. The slides were incubated at RT overnight in a prehybridization buffer mix containing the universal 16S rRNA EUB338 probe coupled to Alexa546 and the *vapB/C-*1 and *vapB/C-*2 probes at a concentration of 50 nM. After three stringent washes in 2× SSC, 1× SSC and 0.5× SSC, the slides were blocked using a blocking reagent (PBS with 1% BSA and 0.5% Triton X-100) for 30 min at RT and then incubated with an anti-DIG Fluorescein Fab fragment (Roche, Mannheim Germany) in a blocking reagent for 1 h at 37°C. Unbound antibody was removed by three washes in PBS with 1% BSA and 0.5% Triton X-100 for 10 min each. The slides were rapidly washed in PBS and MilliQ water before being mounted with DAPI from a ready-to-use solution, ProLong Gold Antifade Reagent (Molecular Probes, Invitrogen, Cergy-Pontoise, France). Following *in situ* hybridization, cells were visualized under a Leica DM2500 upright fluorescence microscope with appropriate filter sets for fluorescein and DAPI fluorescence and an ×100 oil immersion objective. The images were acquired using a cooled (−30°C) DS1-QM (Nikon) black-and-white camera and analyzed using LuciaG software and ImageJ treatment.

### Oligonucleotide FISH probes

The eubacterial probe EUB 338: 5′Alexa 546-GCTGCCTCCCGTAGGAGT [37] and purified PCR-amplified *vapB/C-1* or *vapB/C-2* operons were labeled using a DIG High Prime kit, following the manufacturer's recommendations (Roche, Mannheim Germany).

## Supporting Information

Figure S1 **TA module families.**
(TIF)

Figure S2 **VapB/C protein sequence homology.**
(TIF)

Figure S3 **VapB/C phylogeny.** Neighbor-joining trees are based on concatenated VapC-VapB protein sequences. Taxonomies are highlighted: *Rickettsia* in red, *Cyanobacteria* in green, *Spirochete* in magenta, Proteobacteria other than *Rickettsia* in blue and the rest in black. The accession numbers for each node name can be found in Data S1. Numerals at the end of species names correspond to the number of TA modules found in each species.
(TIF)

Figure S4 **A, Expression of the** ***R. felis vapC-2*** **gene in recombinant yeast cells began an apoptotic response.** **A,** DIC and fluorescence microscopy with Anx-V-FITC and PI staining were performed on yeast spheroblasts carrying the *vapC-2* expression plasmid in media with inducer (Sgal) or without inducer (SD). Yeast spheroblasts prepared from SGal are characteristic of apoptotic cells, with positive annexin staining. Bar scale = 10 µm. **B,** Electron microscopy analysis of *vapC-2* yeast cells grown in SD (i) or Sgal (j) medium. Bar scale = 1 µm. **C**. *In vitro* RNase activity of free *R. felis* VapC-2 protein: The total *E. coli* RNA profiles after incubation reactions with no protein (lane 1), BSA as negative control protein (lane 2), VapC-2 (lane 3), VapB/C-2 complex (lane 4), VapB-2 (lane 5), RNase A as positive control (lane 6).
(PDF)

Data S1 **Tree node name mapping with RefSeq accession and taxonomy data.**
(TIF)

Data S2 **MALDI-TOF Mass Spectrometry and protein Identification.**
(PDF)

Data S3 **MALDI-TOF Mass Spectrometry and protein Identification.**
(PDF)

Table S1 **Gostats functional analysis of differentially expressed genes.**
(PDF)

Table S2 **Primers used in this study.**
(PDF)

Video S1 **Time-lapse videos of L929 cells microinjected with purified** ***R. bellii*** **and** ***R. felis*** **toxin.** The time-lapse video follows the microinjection process and the resulting effects on cells. The white label identifies the microinjected cell. The marked cells show peripheral dynamics, with nuclear retractions and membrane blebbings of cells undergoing apoptosis. The upper left corner displays the time. http://ifr48.timone.univ-mrs.fr/article/toxine-antitoxine/video.zip (http://ifr48.timone.univ-mrs.fr/article/toxine-antitoxine/video.zip).
(DOCX)

Methods S1 (DOCX)
