# Supplementary figures and images for: Effect of Rickettsial Toxin VapC on Its Eukaryotic Host
Source: PLoS One. 2011 Oct 27;6(10):e26528. doi: 10.1371/journal.pone.0026528 (PMC3203148; doi:10.1371/journal.pone.0026528)

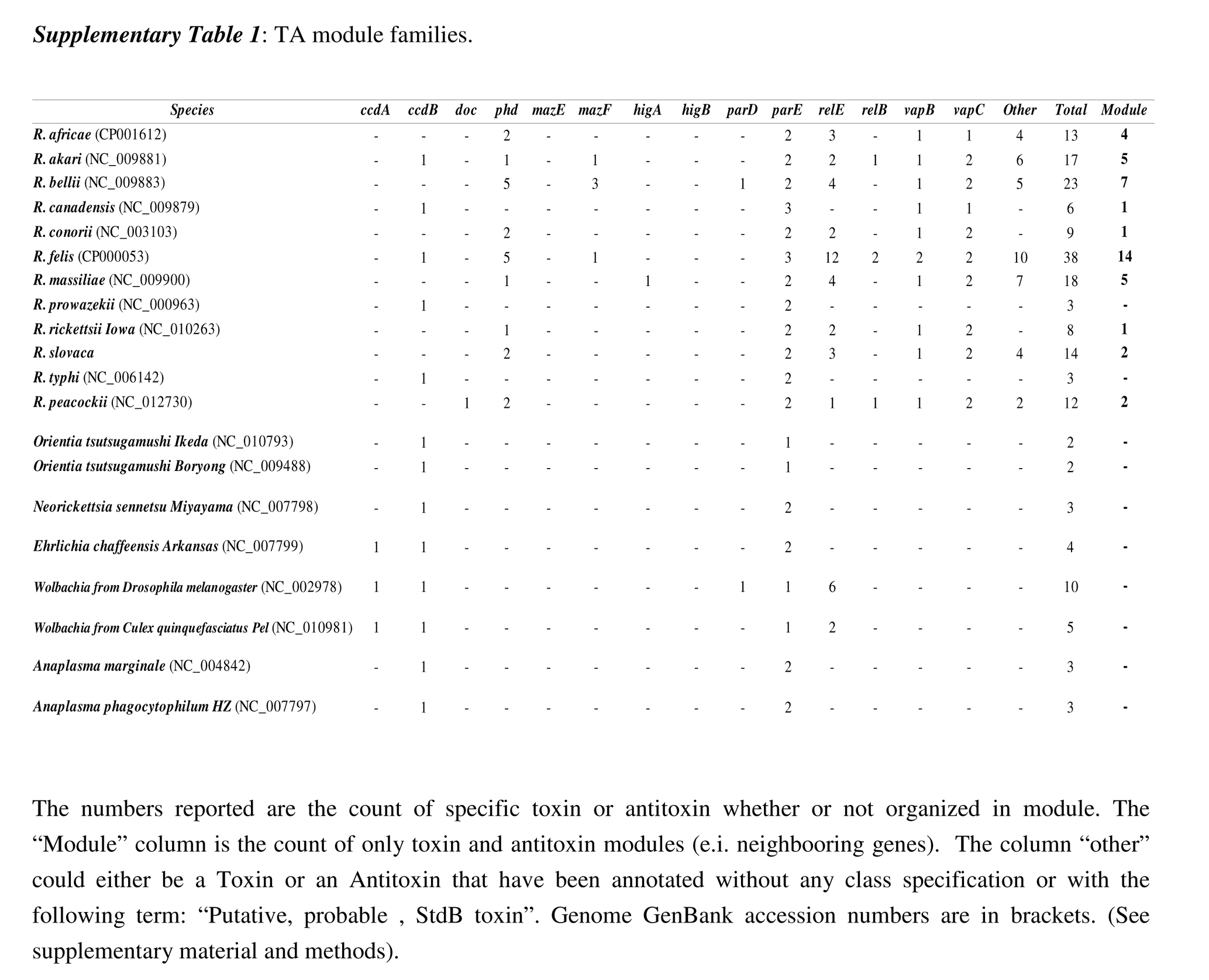

Supplement: Figure S1 — TA module families. (TIF) [file pone.0026528.s001.tif]

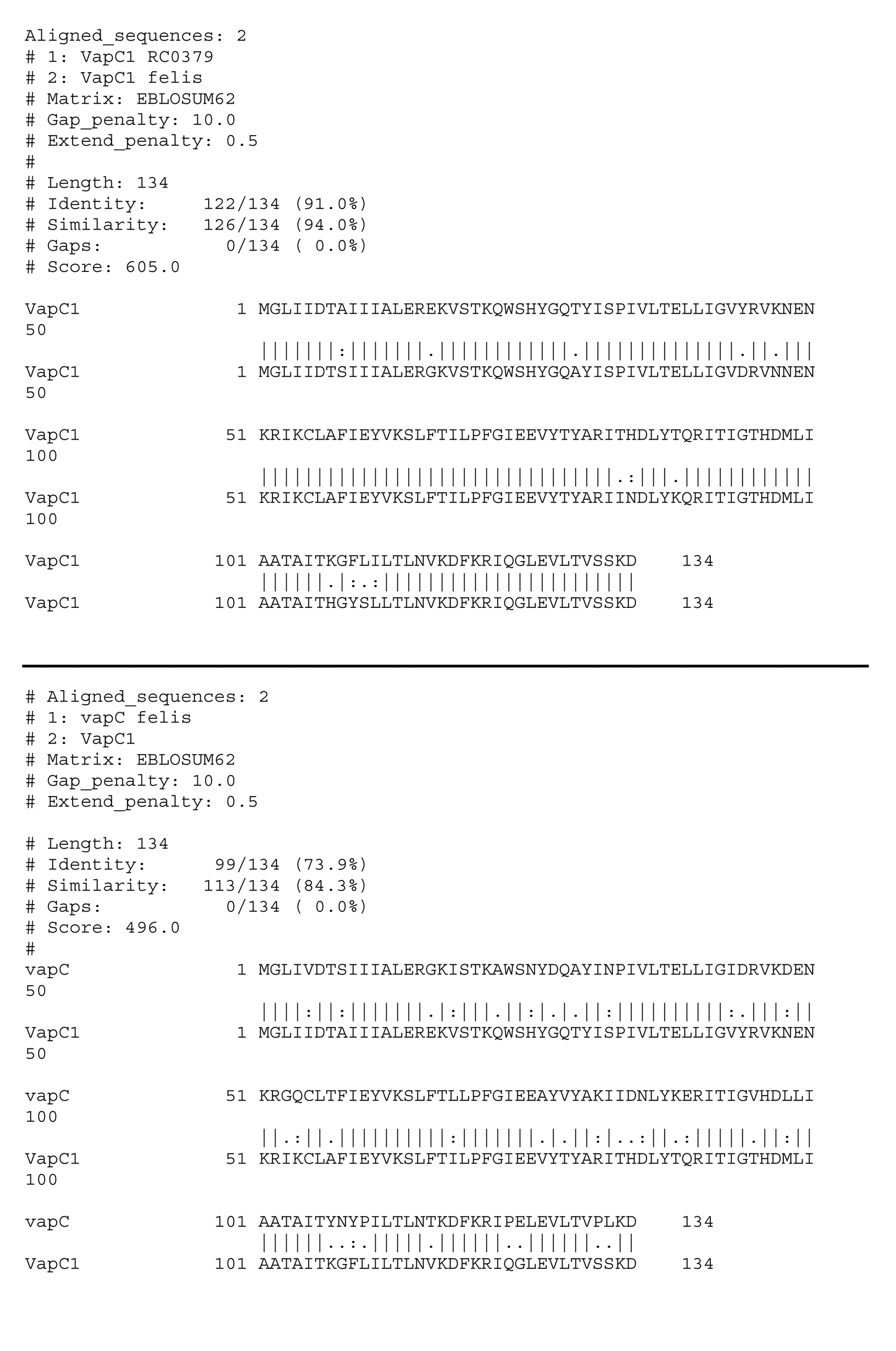

Supplement: Figure S2 — VapB/C protein sequence homology. (TIF) [file pone.0026528.s002.tif]

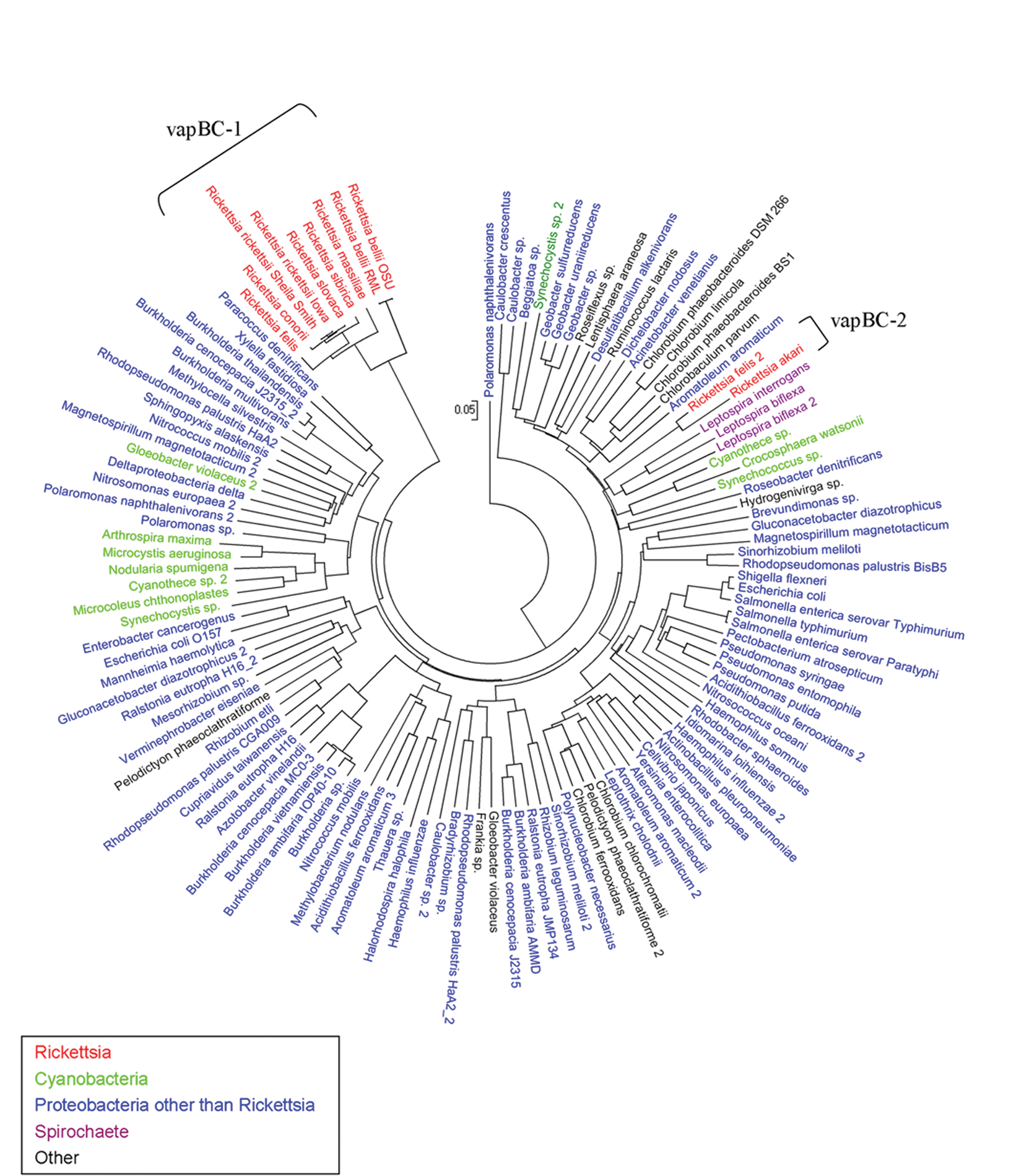

Supplement: Figure S3 — VapB/C phylogeny. Neighbor-joining trees are based on concatenated VapC-VapB protein sequences. Taxonomies are highlighted: Rickettsia in red, Cyanobacteria in green, Spirochete in magenta, Proteobacteria other than Rickettsia in blue and the rest in black. The accession numbers for each node name can be found in Data S1. Numerals at the end of species names correspond to the number of TA modules found in each species. (TIF) [file pone.0026528.s003.tif]

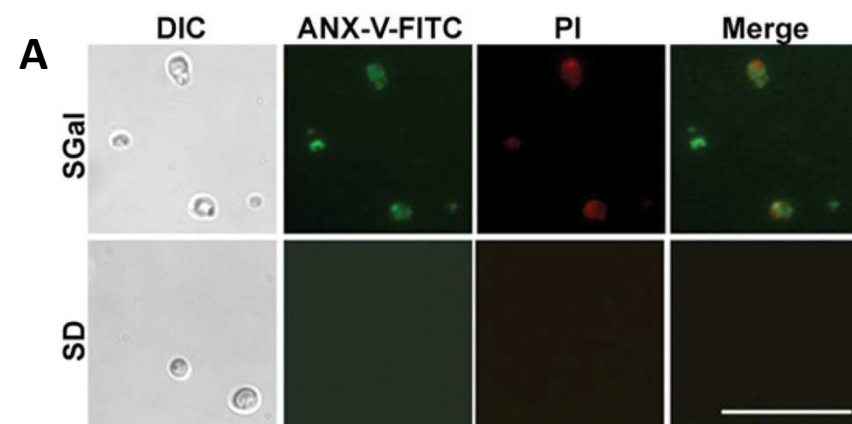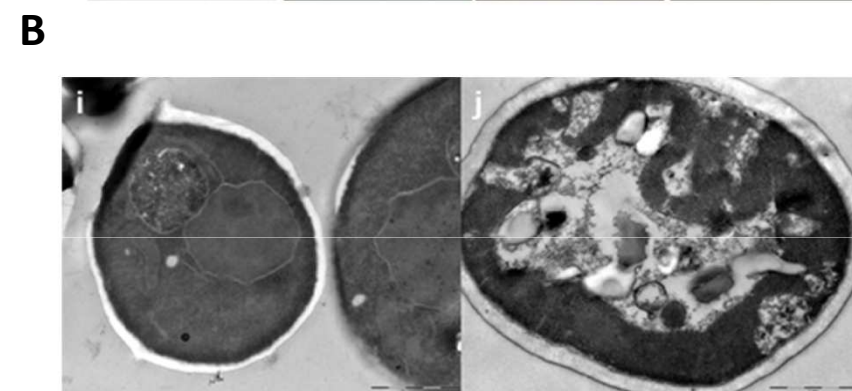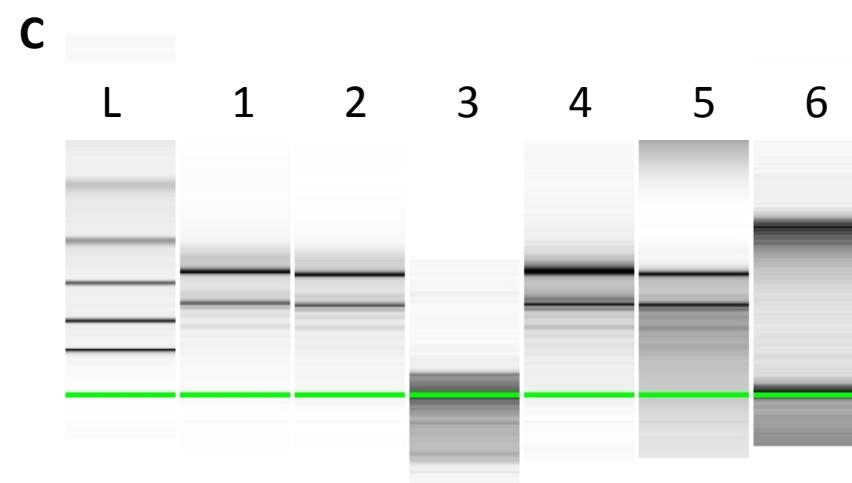

Supplement: Figure S4 — A, Expression of the R. felis vapC-2 gene in recombinant yeast cells began an apoptotic response. A, DIC and fluorescence microscopy with Anx-V-FITC and PI staining were performed on yeast spheroblasts carrying the vapC-2 expression plasmid in media with inducer (Sgal) or without inducer (SD). Yeast spheroblasts prepared from SGal are characteristic of apoptotic cells, with positive annexin staining. Bar scale = 10 µm. B, Electron microscopy analysis of vapC-2 yeast cells grown in SD (i) or Sgal (j) medium. Bar scale = 1 µm. C. In vitro RNase activity of free R. felis VapC-2 protein: The total E. coli RNA profiles after incubation reactions with no protein (lane 1), BSA as negative control protein (lane 2), VapC-2 (lane 3), VapB/C-2 complex (lane 4), VapB-2 (lane 5), RNase A as positive control (lane 6). (PDF) [file pone.0026528.s004.pdf]

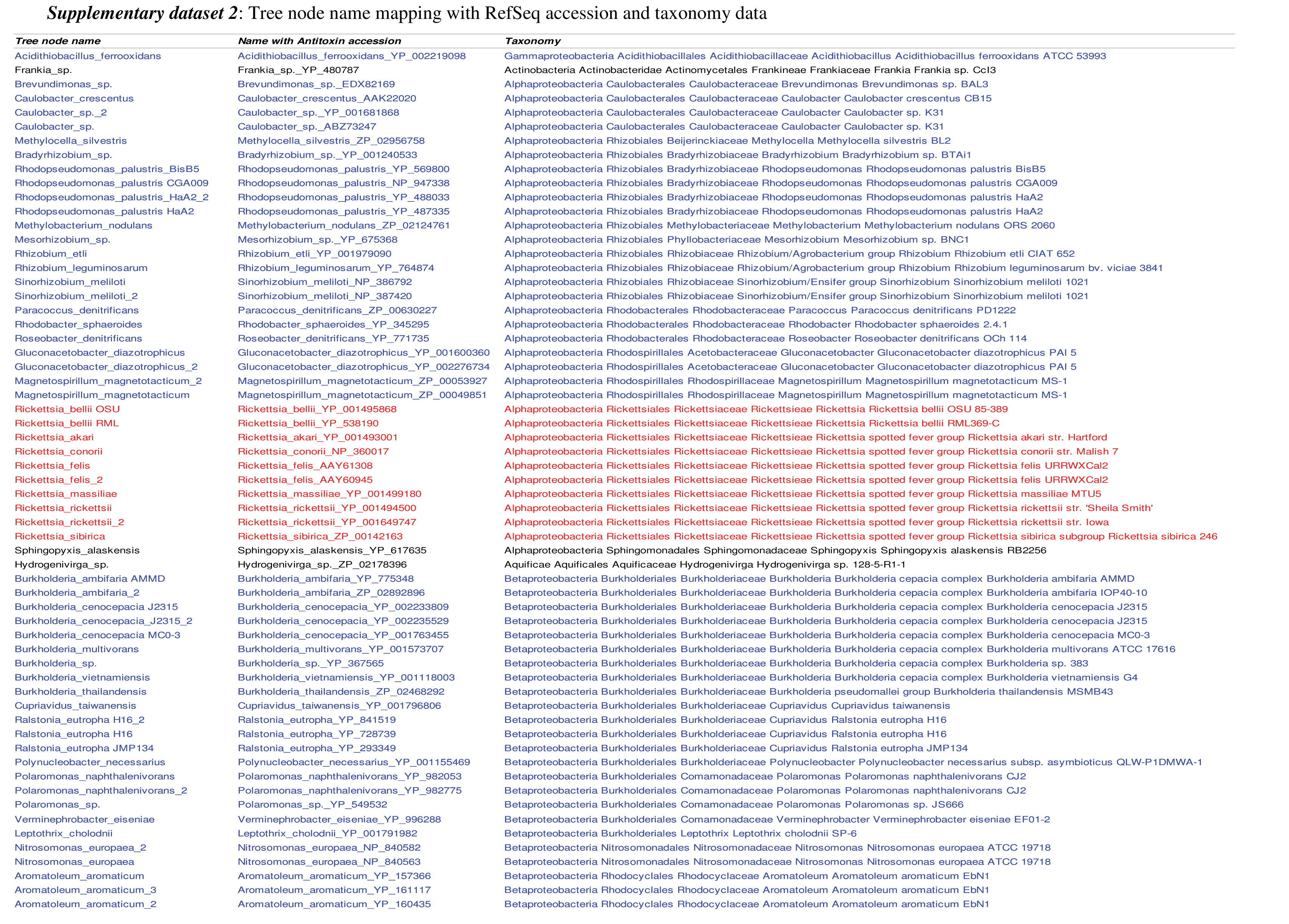

Supplement: Data S1 — Tree node name mapping with RefSeq accession and taxonomy data. (TIF) [file pone.0026528.s005.tif]
