# Supplementary material for: Effect of Rickettsial Toxin VapC on Its Eukaryotic Host
Source: PLoS One. 2011 Oct 27;6(10):e26528. doi: 10.1371/journal.pone.0026528 (PMC3203148; doi:10.1371/journal.pone.0026528)
Supplement: Data S2 — MALDI-TOF Mass Spectrometry and protein Identification. (PDF) [file pone.0026528.s006.pdf]

Spectrum Analysis Report
Date: 08/22/2008 Time: 09:07
FileName: S:\USERS\Christophe\10-07-08\AT\0_N16\1\1SRef\pdata\1\1r

Sequence Name:	>protrefe094-AT	Formula:	
Parentmass:		Mass Error:	
MH+ (mono):	1.008	MH+ (avg):	1.008
Threshold (a.i.):	0.000	Tolerance (Da):	0.200
Number of Peaks:	57	Above Threshold:	
Assigned Peaks:		Not assigned Peaks:	

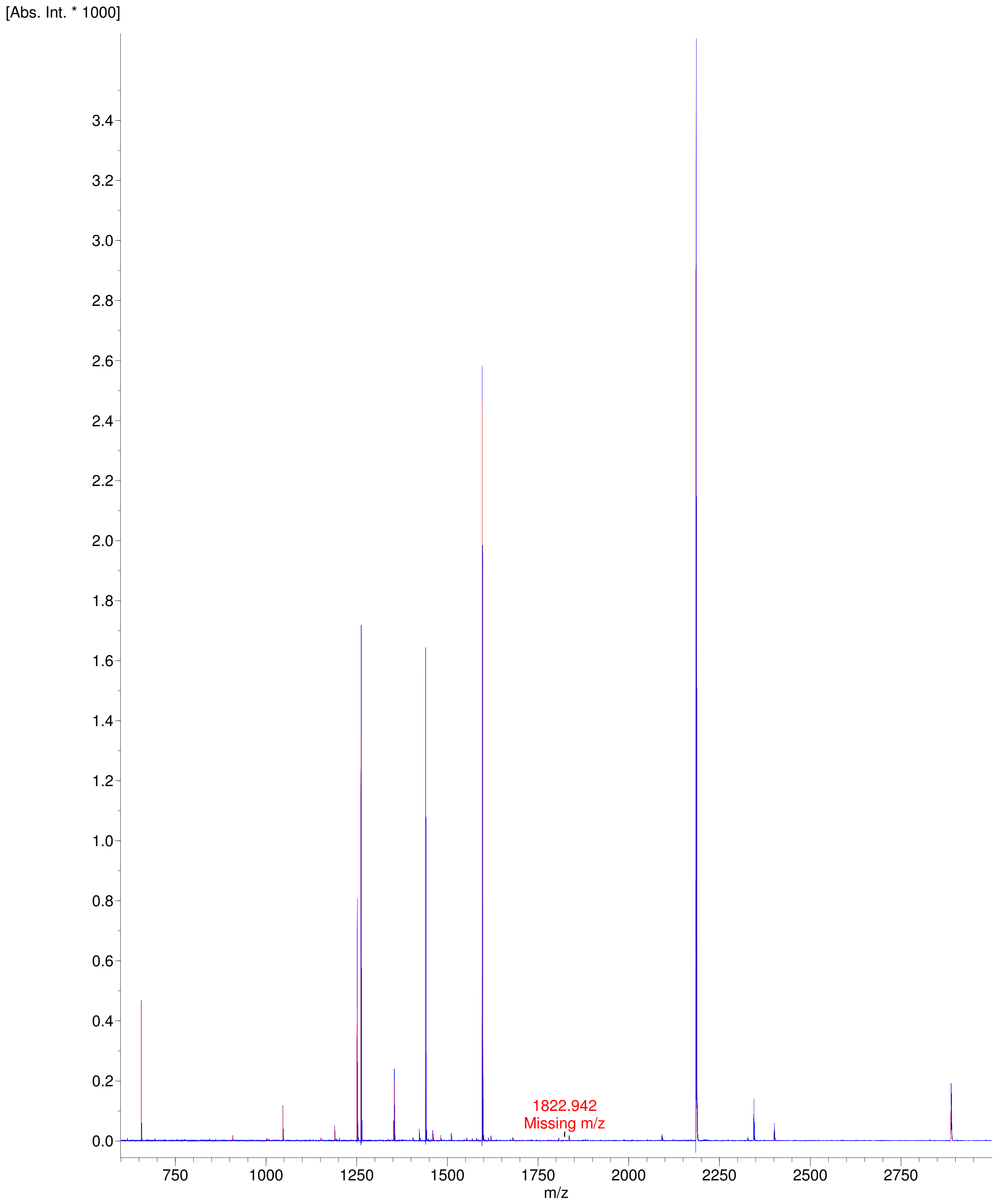

Spectrum Analysis Report

Date: 08/22/2008 Time: 09:07

FileName: S:\USERS\Christophe\10-07-08\AT\0_N16\1\1SRef\pdata\1\1r

Sequence data:

>protrefe094-AT

Intensity Coverage: 30.3 % (3270 cnts)
Sequence Coverage MS/MS: 0.0%
Sequence Coverage MS: 74.4%
pI (isoelectric point): 9.9

```
          10         20         30         40         50         60
MNKAKIFMNG QSQAVRLPKE FRFSVKEVSV IPLGKGIVLQ PLPNSWKDVF QEMAEISSDD
          70         80
IFPEGRKDLP PQKRKYFE
```

Display Parameter:

Sequence Name: >protrefe094-AT
MH+ (avg): 1.008
Tolerance (Da): 0.200
MH+ (mono): 1.008
Threshold (a.i.): 0.000
Number of Peaks: 57

Peaklist:

Peak	Mass	Intensity	Peak	Mass	Intensity
1	610.339	3.421	2	618.355	9.493
3	656.115	422.391	4	672.098	4.491
5	693.629	5.790	6	845.164	5.095
7	908.449	16.076	8	1001.552	8.218
9	1006.859	3.465	10	1008.452	4.277
11	1046.514	101.429	12	1151.415	9.967
13	1189.480	42.820	14	1194.484	6.252
15	1201.573	9.920	16	1250.509	371.289
17	1251.502	433.901	18	1261.589	1305.559
19	1262.619	607.157	20	1336.640	4.259
21	1346.554	3.786	22	1351.631	64.993
23	1353.562	189.028	24	1404.651	10.024
25	1422.647	36.517	26	1439.678	1498.238
27	1447.636	6.160	28	1455.612	4.400
29	1459.598	31.265	30	1481.626	15.072
31	1510.701	22.152	32	1544.656	3.806
33	1553.575	7.497	34	1568.565	6.843
35	1579.678	7.894	36	1595.621	2376.946
37	1612.693	8.642	38	1619.782	10.633
39	1672.671	3.152	40	1679.721	10.015
41	1731.753	2.978	42	1745.787	3.122
43	1805.733	7.866	44	1834.859	13.702
45	1879.881	4.849	46	1986.853	2.793
47	2049.674	3.173	48	2090.836	15.984
49	2120.960	2.854	50	2184.803	2820.326
51	2272.927	2.316	52	2326.980	7.118
53	2343.924	84.921	54	2373.982	1.829
55	2399.984	34.804	56	2829.183	1.837
57	2887.245	95.797			

Matched Sequences:

Unmatched

Peaks

Entries: Meas. M/z Calc. MH+ Int. Dev.(ppm) Range P Sequence

peak 1 610.339 - 3.421 - -
peak 2 618.355 - 9.493 - -
peak 3 656.115 - 422.391 - -
peak 4 672.098 - 4.491 - -
peak 5 693.629 - 5.790 - -
peak 6 845.164 - 5.095 - -
peak 7 908.449 - 16.076 - -
peak 8 1001.552 - 8.218 - -
peak 9 1006.859 - 3.465 - -
peak 10 1008.452 - 4.277 - -
peak 11 1046.514 - 101.429 - -
peak 13 1189.480 - 42.820 - -
peak 14 1194.484 - 6.252 - -
peak 15 1201.573 - 9.920 - -
peak 17 1251.502 - 433.901 - -
peak 18 1261.589 - 1305.559 - -
peak 19 1262.619 - 607.157 - -
peak 20 1336.640 - 4.259 - -
peak 21 1346.554 - 3.786 - -
peak 23 1353.562 - 189.028 - -
peak 24 1404.651 - 10.024 - -
peak 25 1422.647 - 36.517 - -

Copyright © 1999-2003 Bruker Daltonik GmbH

peak 26 1439.678 - 1498.238 - -
peak 27 1447.636 - 6.160 - -
peak 28 1455.612 - 4.400 - -
peak 29 1459.598 - 31.265 - -
peak 30 1481.626 - 15.072 - -
peak 32 1544.656 - 3.806 - -
peak 33 1553.575 - 7.497 - -
peak 34 1568.565 - 6.843 - -
peak 35 1579.678 - 7.894 - -
peak 36 1595.621 - 2376.946 - -
peak 37 1612.693 - 8.642 - -
peak 38 1619.782 - 10.633 - -
peak 39 1672.671 - 3.152 - -
peak 40 1679.721 - 10.015 - -
peak 41 1731.753 - 2.978 - -
peak 42 1745.787 - 3.122 - -
peak 43 1805.733 - 7.866 - -
peak 46 1986.853 - 2.793 - -
peak 47 2049.674 - 3.173 - -
peak 48 2090.836 - 15.984 - -
peak 49 2120.960 - 2.854 - -
peak 51 2272.927 - 2.316 - -
peak 52 2326.980 - 7.118 - -
peak 53 2343.924 - 84.921 - -
peak 54 2373.982 - 1.829 - -
peak 55 2399.984 - 34.804 - -
peak 56 2829.183 - 1.837 - -
peak 57 2887.245 - 95.797 - -

>protrefe094-AT

MNKAKIFMNGQSQAVRLPKEFRFSVKEVSVIPLGKGIVLQPLPNSWKDVFQEMAEISSDDIFPEGRKDLPPQKRKYFE

Digest Results

Entries: Meas. M/z Calc. MH+ Int. Dev.(ppm) Range P Sequence

no peak- 591.328 - - 1 - 5 1 MNKAK
no peak- 1822.941 - - 1 - 16 2 MNKAKIFMNGQSQAVR
no peak- 1449.763 - - 4 - 16 1 AKIFMNGQSQAVR
no peak- 1787.995 - - 4 - 19 2 AKIFMNGQSQAVRLPK
peak 16 1250.509 1250.631 371.289 -98.336 6 - 16 0 IFMNGQSQAVR
no peak- 1588.863 - - 6 - 19 1 IFMNGQSQAVRLPK
no peak- 2021.075 - - 6 - 22 2 IFMNGQSQAVRLPKEFR
no peak- 789.462 - - 17 - 22 1 LPKEFR
no peak- 1250.726 - - 17 - 26 2 LPKEFRFSVK
no peak- 912.494 - - 20 - 26 1 EFRFSVK
peak 44 1834.859 1835.043 13.702 -100.541 20 - 35 2 EFRFSVKEVSVIPLGK
no peak- 1402.830 - - 23 - 35 1 FSVKEVSVIPLGK
no peak- 2735.586 - - 23 - 47 2 FSVKEVSVIPLGKGIVLQPLPNSWK
no peak- 941.567 - - 27 - 35 0 EVSVIPLGK
no peak- 2274.322 - - 27 - 47 1 EVSVIPLGKGIVLQPLPNSWK
peak 22 1351.631 1351.773 64.993 -105.663 36 - 47 0 GIVLQPLPNSWK
peak 50 2184.803 2184.975 2820.326 -79.034 48 - 66 0 DVFQEMAEISSDDIFPEGR
no peak- 2313.070 - - 48 - 67 1 DVFQEMAEISSDDIFPEGRK
no peak- 2991.440 - - 48 - 73 2 DVFQEMAEISSDDIFPEGRKDLPPQK
no peak- 825.483 - - 67 - 73 1 KDLPPQK
no peak- 981.584 - - 67 - 74 2 KDLPPQKR
no peak- 697.388 - - 68 - 73 0 DLPPQK
no peak- 853.489 - - 68 - 74 1 DLPPQKR
no peak- 981.584 - - 68 - 75 2 DLPPQKRK
no peak- 742.388 - - 74 - 78 2 RKYFE
no peak- 586.287 - - 75 - 78 1 KYFE

>protrefe95-tox

MIYMLDTNICVYAINKHPDSYYNNLELLAKNNTIAISSIVLAELQYGVSKSKKKEQNQSKLDIFLSRLEIIDFSAKCTFYYGELRTELEQKGLIIGNND
LLIASHAIAENATLVTNNIKEFKRIPNLILENWDK

Digest Results

Entries: Meas. M/z Calc. MH+ Int. Dev.(ppm) Range P Sequence

no peak- 1904.932 - - 1 - 16 0 MIYMLDTNICVYAINK
no peak- 1676.828 - - 17 - 30 0 HPDSYYNNLELLAK
no peak- 2120.160 - - 31 - 50 0 NNTIAISSIVLAELQYGVSK
no peak- 2335.287 - - 31 - 52 1 NNTIAISSIVLAELQYGVSKSK
no peak- 2463.382 - - 31 - 53 2 NNTIAISSIVLAELQYGVSKSKK
no peak- 989.537 - - 53 - 60 2 KKEQNQSK
no peak- 861.442 - - 54 - 60 1 KEQNQSK
no peak- 1705.923 - - 54 - 67 2 KEQNQSKLDIFLSR
no peak- 733.348 - - 55 - 60 0 EQNQSK
no peak- 1577.828 - - 55 - 67 1 EQNQSKLDIFLSR
no peak- 2594.382 - - 55 - 76 2 EQNQSKLDIFLSRLEIIDFSAK
no peak- 863.499 - - 61 - 67 0 LDIFLSR

peak 45 1879.881 1880.053 4.849 -91.508 61 - 76 1 LDIFLSRLEIIDFSAK
no peak- 1035.572 - - 68 - 76 0 LEIIDFSAK
no peak- 2168.073 - - 68 - 85 1 LEIIDFSAKCTFYYGELR
no peak- 2896.444 - - 68 - 91 2 LEIIDFSAKCTFYYGELRTELEQK
peak 12 1151.415 1151.519 9.967 -91.288 77 - 85 0 CTFYYGELR
peak 45 1879.881 1879.889 4.849 -4.603 77 - 91 1 CTFYYGELRTELEQK
no peak- 747.388 - - 86 - 91 0 TELEQK
no peak- 2902.600 - - 92 - 119 0 GLIIGNNDLLIASHAIAENATLVTNNIK
no peak- 579.325 - - 120 - 123 1 EFKR
no peak- 1915.044 - - 120 - 134 2 EFKRIPNLILENWDK
peak 31 1510.701 1510.838 22.152 -91.009 123 - 134 1 RIPNLILENWDK
no peak- 1354.737 - - 124 - 134 0 IPNLILENWDK
