# Supplementary material for: Effect of Rickettsial Toxin VapC on Its Eukaryotic Host
Source: PLoS One. 2011 Oct 27;6(10):e26528. doi: 10.1371/journal.pone.0026528 (PMC3203148; doi:10.1371/journal.pone.0026528)
Supplement: Table S1 — Gostats functional analysis of differentially expressed genes. (PDF) [file pone.0026528.s008.pdf]

Genes implicated in apoptosis process

ProbeID	UniGeneID	Gene Name	Fold Change
A_32_P97489	Hs.492407	tyrosine 3-monooxygenase	0.715582973988485
A_23_P89431	Hs.303649	chemokine (C-C motif) ligand 2	0.857641562607039
A_24_P262395	Hs.195740	apoptosis antagonizing transcription factor	0.655047199309045
A_23_P22957	Hs.136309	SH3-domain GRB2-like endophilin B1	0.818065473490335
A_24_P336759	Hs.632486	myeloid cell leukemia sequence 1 (BCL2-related)	0.649057100975724
A_23_P63896	Hs.244139	Fas (TNF receptor superfamily, member 6)	0.76437987128329
A_23_P118815	Hs.514527	baculoviral IAP repeat-containing 5 (survivin)	0.907992554377479
A_23_P26084	Hs.32148	selenoprotein S	0.693809934733732

Gene implicated in anti apoptotic process

ProbeID	UniGeneID	Gene Name	Fold Change
A_23_P382775	Hs.467020	BCL2 binding component 3	1.21739230375997
A_23_P120883	Hs.517581	heme oxygenase (decycling) 1	1.74810781402233
A_23_P358944	Hs.526464	promyelocytic leukemia	1.52136157568064
A_23_P125990	Hs.194333	E2F transcription factor 2	1.16821971241019
A_24_P281101	Hs.431048	c-abl oncogene 1, receptor tyrosine kinase	1.43022203681482
A_23_P207850	Hs.438292	tensin 4	1.29765247015053
A_23_P167256	Hs.12420	PHD finger protein 17	1.39039477020604

Significantly over-represented GO biological processes of up-regulated genes

GOBPID	Pvalue	OddsRatio	ExpCount	Count	Size	Term
GO:0008630	0.000	29.586	0	4	19	DNA damage response, signal transduction resulting in induction of apoptosis
GO:0008629	0.000	14.776	0	4	34	induction of apoptosis by intracellular signals
GO:0042770	0.001	10.802	0	4	45	DNA damage response, signal transduction
GO:0002448	0.002	43.655	0	2	7	mast cell mediated immunity
GO:0043303	0.002	43.655	0	2	7	mast cell degranulation
GO:0042771	0.002	36.376	0	2	8	DNA damage response, signal transduction by p53 class mediator resulting in induction of apoptosis
GO:0045576	0.002	36.376	0	2	8	mast cell activation
GO:0032507	0.002	12.680	0	3	29	maintenance of protein location in cell
GO:0043299	0.003	31.177	0	2	9	leukocyte degranulation
GO:0051651	0.003	10.986	0	3	33	maintenance of location in cell
GO:0045185	0.004	10.298	0	3	35	maintenance of protein location
GO:0002444	0.004	24.245	0	2	11	myeloid leukocyte mediated immunity
GO:0032386	0.006	9.151	0	3	39	regulation of intracellular transport
GO:0045055	0.006	19.834	0	2	13	regulated secretory pathway

Significantly over-represented GO biological processes of low-regulated genes

GOBPID	Pvalue	OddsRatio	ExpCount	Count	Size	Term
GO:0051301	0.000	4.043	3	12	234	cell division
GO:0007051	0.000	19.351	0	4	19	spindle organization and biogenesis
GO:0000278	0.000	3.329	5	15	355	mitotic cell cycle
GO:0000279	0.000	3.651	4	13	280	M phase
GO:0007067	0.000	4.118	3	11	210	mitosis
GO:0000087	0.000	4.076	3	11	212	M phase of mitotic cell cycle
GO:0022402	0.000	2.880	6	17	464	cell cycle process
GO:0022403	0.000	3.214	5	14	341	cell cycle phase
GO:0006412	0.000	3.035	5	15	387	translation
GO:0006916	0.002	3.919	2	8	158	anti-apoptosis
GO:0031326	0.003	3.879	2	7	139	regulation of cellular biosynthetic process
GO:0043066	0.004	3.107	3	9	222	negative regulation of apoptosis
GO:0043069	0.004	3.063	3	9	225	negative regulation of programmed cell death
GO:0030705	0.005	4.011	2	6	115	cytoskeleton-dependent intracellular transport
GO:0008654	0.007	4.482	1	5	86	phospholipid biosynthetic process
GO:0009987	0.007	1.839	150	162	10819	cellular process
GO:0006643	0.008	2.957	3	8	206	membrane lipid metabolic process
GO:0007018	0.009	4.171	1	5	92	microtubule-based movement
GO:0045429	0.010	15.951	0	2	11	positive regulation of nitric oxide biosynthetic process
