# Supplementary material for: Effect of Rickettsial Toxin VapC on Its Eukaryotic Host
Source: PLoS One. 2011 Oct 27;6(10):e26528. doi: 10.1371/journal.pone.0026528 (PMC3203148; doi:10.1371/journal.pone.0026528)
Supplement: Table S2 — Primers used in this study. (PDF) [file pone.0026528.s009.pdf]

Primer name	Target	Sense	Sequence	Fragment size (bp)	Used for
NterRF0094	*R.felis-vapB-2*	forward	*GGGGACAAGTTTGTACAAAAAAGCAGGCTTAGAAAACCTGTACTTCCAGGGT*AATAAAGCAAAAATATTTATGAATG	320	Gateway cloning system in *E.coli*
CterRF0094	*R.felis-vapB-2*	reverse	*GGGGACCACTTTGTACAAGAAAGCTGGGTCTTATTA*TTCAAAATATTTCCTTTTTTGTGGT		
NterRF0095	*R.felis-vapC-2*	forward	*GGGGACAAGTTTGTACAAAAAAGCAGGCTTAGAAAACCTGTACTTCCAGGGT*ATCTACATGCTTGACACTAATATTT	488	Gateway cloning system in *E.coli*
CterRF0095	*R.felis-vapC-2*	reverse	*GGGGACCACTTTGTACAAGAAAGCTGGGTCTTATTA*TTTATCCCAATTTTCTAAAATTAAA		
SRFE94	*R.felis vapB-2*	forward	*CGACTCACTATAGGGAATATTA*ATGAATAAAGCAAAAATATTTATG	280	Yeast recombination cloning
RRFE94	*R.felis*	reverse	*CCAGCACACTGGCGGCCGTTACTA*TTCAAAATATTTCCTTTTTTGTGGTGG		
SRFE95	*R.felis vapC-2*	forward	*CGACTCACTATAGGGAATATTAAGCTA*TGATCTACATGCTTGACAC	452	Yeast recombination cloning
RRFE95	*R.felis vapC-2*	reverse	*CCAGCACACTGGCGGCCGTTACTA*TTTATCCCAATTTTCTAAAATTAAATTAGG		
Nco94	*R.felis vapB-2*	forward	GCAACTCCATGGATAAAGCAAAAATATTTATG	254	PCR screen RT-PCR
Sma94		reverse	AGTTGCCCCGGGTTCAAAATATTTCCTTTTTTGTGG		
S-95RF	*R.felis vapC-2*	forward	GCAAAATCAAAGCAAATTGGA	178	PCR screen RT-PCR
R-95RF		reverse	AACTAAAGTTGCGTTTTCAGCTA		
S-RBvapB1	*R.bellii vapB1*	forward	*GGGGACAAGTTTGTACAAAAAAGCAGGCTTAGAAAACCTGTACTTCCAGGGT*ATGAATAAATGGCAATTACACGAAG		Gateway cloning system in *E.coli*
R-RB-vapB1	*R.bellii vapB1*	reverse	*GGGGACCACTTTGTACAAGAAAGCTGGGTCTTATTA*TTCAAAATCTCTTGCCTTTCCTTG	246	Gateway cloning system in *E.coli*
S-RBvapC1	*R.bellii-vapC1*	forward	*GGGGACAAGTTTGTACAAAAAAGCAGGCTTAGAAAACCTGTACTTCCAGGGT*ATGAAATATTTATTAGATACTAATG		Gateway cloning system in *E.coli*
R-RB vapC1	*R.bellii-vapC1*	reverse	*GGGGACCACTTTGTACAAGAAAGCTGGGTCTTATTA*GTAACTAATATCATATTGTTTTGT		Gateway cloning system in *E.coli*

*sequences *in italic* correspond to homologous sequences of recombination sites of cloning vectors
