# Supplementary material for: Effect of Rickettsial Toxin VapC on Its Eukaryotic Host
Source: PLoS One. 2011 Oct 27;6(10):e26528. doi: 10.1371/journal.pone.0026528 (PMC3203148; doi:10.1371/journal.pone.0026528)
Supplement: Video S1 — Time-lapse videos of L929 cells microinjected with purified R. bellii and R. felis toxin. The time-lapse video follows the microinjection process and the resulting effects on cells. The white label identifies the microinjected cell. The marked cells show peripheral dynamics, with nuclear retractions and membrane blebbings of cells undergoing apoptosis. The upper left corner displays the time. http://ifr48.timone.univ-mrs.fr/article/toxine-antitoxine/video.zip (http://ifr48.timone.univ-mrs.fr/article/toxine-antitoxine/video.zip). (DOCX) [file pone.0026528.s010.docx]

**S7: Time-lapse videos of L929 cells** **microinjected with purified *R. bellii* and *R. felis* toxin.** The time-lapse video follows the microinjection process and the resulting effects on cells. The white label identifies the microinjected cell. The marked cells show peripheral dynamics, with nuclear retractions and membrane blebbings of cells undergoing apoptosis. The upper left corner displays the time.

http://ifr48.timone.univ-mrs.fr/article/toxine-antitoxine/video.zip

(<http://ifr48.timone.univ-mrs.fr/article/toxine-antitoxine/video.zip>)
