# Supplementary material for: Effect of Rickettsial Toxin VapC on Its Eukaryotic Host
Source: PLoS One. 2011 Oct 27;6(10):e26528. doi: 10.1371/journal.pone.0026528 (PMC3203148; doi:10.1371/journal.pone.0026528)
Supplement: Methods S1 — (DOCX) [file pone.0026528.s011.docx]

**Methods S1:**

**Microarray data processing and analysis:** Raw data from the Agilent feature extraction software were preprocessed with background subtraction and quantile normalization. This pre-treatment was performed with the bioconductor limma package Agi4x44PreProcess (<http://www.bioconductor.org/packages/2.3/bioc/html/> [Agi4x44PreProcess.html](http://www.bioconductor.org/packages/2.3/bioc/html/Agi4x44PreProcess.html)). Data sets were then pre-analyzed using Significance Analysis of Microarrays (SAM)[1]. SAM analysis scores the statistical significance of each gene and estimates the false discovery rate. To segment the microarray data into biologically significant groups, we associated all genes with Gene Ontology terms. This functional analysis was carried out with the Gostats [2] and hgug4112a.db annotation packages (<http://www.bioconductor.org/packages/2.4/data/annotation/html/> [hgug4112a.db.html](http://www.bioconductor.org/packages/2.4/data/annotation/html/hgug4112a.db.html)), which allow tests for over-representation of GO terms.

**Molecular cloning:** The cloning of *R. felis vapB1, vapC1, vapB2*, and *vapC2* genes and *R. bellii* *vapB1* and *vapC1* for expression in *E. coli* was performed by recombination in the Gateway system (Invitrogen, Ltd., Paisley, U.K.). Each primers contain the 5’ (attB1) and 3’ (attB2) recombination sites at the 5’ end, respectively (Table S8). All constructs were cloned following the BP reaction in pDONR 201 and transferred by the LR reaction to pDest17 (Invitrogen, Ltd., Paisley, U.K.) and pETG20A as destination vectors. With the construct in pDest17, a His tag is fused on the N terminal of the proteins. With this destination vector, the 5’ end of the *Rickettsia* ORF is fused to a thioredoxin protein, a His tag and a cleavage site specific to the protease TEV. All constructions were confirmed by sequencing, only the *R. felis* VapC-2 presented a mutation R85H. In the case of the *Rickettsia* operons and VapBC constructs, only VapB contains a His tag, and therefore only VapC in complex with VapB will co-purify. The purified plasmids were used to transform the *E. coli* strains Rosetta (DE3) *pLysS* (Novagen, Darmstadt, Germany) [3–5]

**Expression of *R. felis genes* in heterologous host:** Bacterial growth curve detection were done with recombinant Rosetta (DE3) *pLysS* cells grown in 24-well deep-well plates in triplicate in LB medium at 37°C with 600 rpm agitation for 400 min. We took OD readings at 600 nm every 30 min using a 96-well plate reader. Yeast Strains were grown aerobically at 30°C in SD medium until they reached the exponential phase before diluted in fresh SD or Sgal media. Growth curves were recorded by allowing the cells to grow at 30°C and monitoring A_600 nm_ for 30 hours. Yeast survival assays were performed in triplicate. Briefly, 0.3 A_600 nm_ of yeast cells was serially diluted and 20 µl was cultivated in SD or Sgal 96-well plates. The number of surviving colonies was determined under a microscope in the last positive well after 3 days at 30°C. Yeast division and nuclear fragmentation of yeast cells was analyzed after DAPI staining (Invitrogen Ltd., Paisley, U.K.). To analyze yeast apoptosis, Anx-V-FITC and propidium iodide (PI) (AbCys, Paris, France) staining were performed on spheroblasts as described previously[6]. Cells treated with acetic acid a classical apoptosis inducer in yeast cells were done in parallel experiments[7]. Ultrastructural analysis of yeast cells was performed on samples fixed in 2% formaldehyde and 1.5% glutaraldehyde diluted in 0.1 M potassium phosphate solution (pH 7.2) and then dehydrated through a graded ethanol series and embedded in Epon 812 resin (Sigma Aldrich, France).

Reference List

1. Tusher VG, Tibshirani R, Chu G (2001) Significance analysis of microarrays applied to the ionizing radiation response. Proc Natl Acad Sci U S A 98: 5116-5121. 10.1073/pnas.091062498 [doi];091062498 [pii].

2. Falcon S, Gentleman R (2007) Using GOstats to test gene lists for GO term association. Bioinformatics 23: 257-258. btl567 [pii];10.1093/bioinformatics/btl567 [doi].

3. Berrow NS, Bussow K, Coutard B, Diprose J, Ekberg M, Folkers GE, Levy N, Lieu V, Owens RJ, Peleg Y, Pinaglia C, Quevillon-Cheruel S, Salim L, Scheich C, Vincentelli R, Busso D (2006) Recombinant protein expression and solubility screening in Escherichia coli: a comparative study. Acta Crystallogr D Biol Crystallogr 62: 1218-1226. S0907444906031337 [pii];10.1107/S0907444906031337 [doi].

4. Graslund S, Nordlund P, Weigelt J, Hallberg BM, Bray J, Gileadi O, Knapp S, Oppermann U, Arrowsmith C, Hui R, Ming J, dhe-Paganon S, Park HW, Savchenko A, Yee A, Edwards A, Vincentelli R, Cambillau C, Kim R, Kim SH, Rao Z, Shi Y, Terwilliger TC, Kim CY, Hung LW, Waldo GS, Peleg Y, Albeck S, Unger T, Dym O, Prilusky J, Sussman JL, Stevens RC, Lesley SA, Wilson IA, Joachimiak A, Collart F, Dementieva I, Donnelly MI, Eschenfeldt WH, Kim Y, Stols L, Wu R, Zhou M, Burley SK, Emtage JS, Sauder JM, Thompson D, Bain K, Luz J, Gheyi T, Zhang F, Atwell S, Almo SC, Bonanno JB, Fiser A, Swaminathan S, Studier FW, Chance MR, Sali A, Acton TB, Xiao R, Zhao L, Ma LC, Hunt JF, Tong L, Cunningham K, Inouye M, Anderson S, Janjua H, Shastry R, Ho CK, Wang D, Wang H, Jiang M, Montelione GT, Stuart DI, Owens RJ, Daenke S, Schutz A, Heinemann U, Yokoyama S, Bussow K, Gunsalus KC (2008) Protein production and purification. Nat Methods 5: 135-146. nmeth.f.202 [pii];10.1038/nmeth.f.202 [doi].

5. Studier FW (2005) Protein production by auto-induction in high density shaking cultures. Protein Expr Purif 41: 207-234.

6. Madeo F, Frohlich E, Frohlich KU (1997) A yeast mutant showing diagnostic markers of early and late apoptosis. J Cell Biol 139: 729-734.

7. Ludovico P, Sousa MJ, Silva MT, Leao C, Corte-Real M (2001) Saccharomyces cerevisiae commits to a programmed cell death process in response to acetic acid. Microbiology 147: 2409-2415.
